# Increased Resting-State Functional Connectivity in Obese Adolescents; A Magnetoencephalographic Pilot Study

**DOI:** 10.1371/journal.pone.0002827

**Published:** 2008-07-30

**Authors:** Kim T. E. Olde Dubbelink, Abraham Felius, Jeroen P. A. Verbunt, Bob W. van Dijk, Henk W. Berendse, Cornelis J. Stam, Henriette A. Delemarre-van de Waal

**Affiliations:** 1 Department of Paediatric Endocrinology, VU University Medical Center, Amsterdam, The Netherlands; 2 Department of Physics and Medical Technology, VU University Medical Center, Amsterdam, The Netherlands; 3 Department of Clinical Neurophysiology, Institute for Clinical and Experimental Neurosciences, VU University Medical Center, Amsterdam, The Netherlands; Columbia University, United States of America

## Abstract

**Background:**

Obesity is not only associated with metabolic abnormalities, but also with cognitive dysfunction and changes in the central nervous system. The present pilot study was carried out to investigate functional connectivity in obese and non-obese adolescents using magnetoencephalography (MEG).

**Methodology/Principal Findings:**

Magnetoencephalographic recordings were performed in 11 obese (mean BMI 38.8±4.6 kg/m^2^) and 8 lean (mean BMI 21.0±1.5 kg/m^2^) female adolescents (age 12–19 years) during an eyes-closed resting-state condition. From these recordings, the synchronization likelihood (SL), a common method that estimates both linear and non-linear interdependencies between MEG signals, was calculated within and between brain regions, and within standard frequency bands (delta, theta, alpha1, alpha2, beta and gamma). The obese adolescents had increased synchronization in delta (0.5–4 Hz) and beta (13–30 Hz) frequency bands compared to lean controls (*P*_delta total_ = 0.001; *P*_beta total_ = 0.002).

**Conclusions/Significance:**

This study identified increased resting-state functional connectivity in severe obese adolescents. Considering the importance of functional coupling between brain areas for cognitive functioning, the present findings strengthen the hypothesis that obesity may have a major impact on human brain function. The cause of the observed excessive synchronization is unknown, but might be related to disturbed motivational pathways, the recently demonstrated increase in white matter volume in obese subjects or altered metabolic processes like hyperinsulinemia. The question arises whether the changes in brain structure and communication are a dynamic process due to weight gain and whether these effects are reversible or not.

## Introduction

The accumulation of body fat in obesity is associated with several metabolic abnormalities including hyperglycemia, dyslipidemia and hypertension[1]. These metabolic disturbances predispose to diseases like type 2 diabetes, myocardial infarction, stroke and cancer[2]–[4]. It is less well known that obesity is also associated with changes in the central nervous system. Epidemiological studies suggest a link between increased body weight and certain degenerative brain diseases. Weight gain is linked to Alzheimer's disease and other forms of dementia[5], [6], and an association between obesity and Parkinson's disease has been established as well[7], [8]. It has therefore been suggested that obesity may lead to cognitive changes[9]. Overweight and obesity in childhood and adolescence are associated with a high risk to be obese in adulthood[10]. Children and adolescents therefore deserve special attention as a potentially treatable group, in order to prevent weight gain and its potentially devastating consequences.

It has been demonstrated that motivation and control of food intake are regulated by the human brain, and in particular by hypothalamic regions, as well as limbic-frontal connectivity[11]. Those areas serve the human reward system, in which reinforcement learning plays an important role to establish human behaviour[12]. Food is one of the very strong rewards, and patterns of neural activity related to food motivation are probably already established in childhood[13]. Those patterns may however be modulated in less salutary ways that can lead to eating disorders and threaten health, as in obesity[14], [15]. In this way, obesity is comparable to addiction[16].

Functionally, oscillations are a prominent feature of neuronal activity. Oscillatory brain communication can be studied best by using magnetoencephalography (MEG). This non-invasive technique enables the magnetic fields, generated by electrical activity in the brain, to be measured very accurately. Therefore, MEG is quite suitable to study normal as well as pathological communication processes in the human brain. The synchronization of oscillations, which reflects the temporally precise interaction of neural activities, is a generally accepted mechanism for neural communication[17], [18]. Often referred to as ‘functional connectivity’, statistical correlations between oscillatory brain activity of different brain regions are regarded as an index of functional interactions between these brain regions[19]. Synchronization changes (increases as well as decreases) may occur in different frequency bands and can be studied by different methods. Synchronization likelihood (SL) is a very general method that estimates both linear and non-linear interdependencies in MEG signals from different channels[20].

We postulate that obesity is associated with alterations in cortical communication processes. The present pilot study was undertaken to investigate whether there might be a difference in resting-state functional connectivity, as measured using MEG and SL, between obese en non-obese subjects.

## Methods

### Subjects

Eleven obese female adolescents, according to the international criteria for obesity[21], were recruited at the obesity clinic in the outpatient ward of the department of paediatrics, VU University medical center, Amsterdam. The obese adolescents underwent an oral glucose tolerance test before entering the study. This revealed, as expected in this group with severe obesity, disturbances in glucose homeostasis. Fasting glucose was normal in all patients (mean 4.9 mmol/l, SD 0.4 mmol/l, range 4.2–5.2 mmol/l), while steady state beta cell function (%B,) and insulin sensitivity (%S) estimated by The Homeostasis Model Assessment (HOMA) were impaired in 10 out of 11 obese adolescents. Beta cell function was increased (%B mean 225%, SD 88%, range 87%–348%), while insulin sensitivity was decreased (%S mean 46%, SD 52%, range 14%–198%) resulting in an increased insulin resistance (HOMA IR mean 3.7, SD 2.1, range 0.5 to 7.0).

Eight healthy controls with a BMI from 25^th^ till 75^th^ percentile were recruited among age, sex, pubertal stage and education matched peers of the obese participants.

Subjects with a chronic somatic or neurological disease, a body weight above 130 kg, or claustrophobia were excluded from participation. Subjects with metal braces or other irremovable metal devices were also excluded, since these devices interfere with the recording of the MEG signals. After written and oral explanation of the study, the subjects and their parents signed the informed consent. This study was approved by the medical ethics committee at the VU University Medical Centre, and conducted according to the principles of the declaration of Helsinki (version 2004).

### Procedures

In order to create equal circumstances at the time of the measurements, all appointments for data acquisition were planned in the morning. Participants scheduled early in the morning (at 9.00 AM or 10.00 AM) were instructed to attend the recording session after an overnight fasting state. If their appointment was late in the morning (at 11.00 or 12.00 AM) they were instructed to have a light breakfast (cup of tea without sugar and a piece of toast) at least three hours prior to the recording session.

On arrival at the clinic, weight and height of the subjects were measured. In addition an explanation of the procedure was given.

MEG registrations were performed in a resting-state, eyes-closed condition. During the MEG recordings, subjects were instructed to stay awake and to sit as still and relaxed as possible for 5 minutes with their eyes closed.

### Physiological measures/MEG recordings

Magnetic fields were recorded while subjects were seated inside a three-layer magnetically shielded room (Vacuumschmelze GmbH, Hanau, Germany) using a 151 channel whole-head MEG system (CTF systems Inc., Vancouver, Canada). The sensors were uniformly distributed on the helmet surface with mean spacing of 3.1 cm. The SQUID sensors were 1^st^ order axial hardware gradiometers with a 2 cm coil diameter and a 5 cm baseline. In addition there was an array of 36 reference sensors (12 magnetometers and 24 gradiometers) that was used for noise cancellation by means of software formation of 3^rd^ order synthetic gradiometers. A 3^rd^ order software gradient was used with a recording passband of DC–200 Hz and a sample rate of 625 Hz. Powerline noise was removed by 4^th^ order low pass anti-aliasing filter at 200 Hz and notch filters at 50, 100 and 150 Hz. The total system noise level was less then 10 fT rms/sqrt(Hz). At the beginning and end of each condition the head position relative to the coordinate system of the helmet was recorded by leading small AC currents through three head position coils attached to the left and right pre-auricular points and the nasion on the subjects head.

### Synchronization likelihood

The synchronization likelihood is a general measure of the statistical interdependencies between two time series that was described in detail previously[20] and will be briefly summarized here. We assume two dynamic systems, for instance neural networks, designated X and Y. From both systems time series *x_i_* and *y_i_*, for instance EEG or MEG signals, are recorded. The general problem is to infer functional interactions between X and Y from *x_i_* and *y_i_*. Usually it is assumed that the more *x_i_* and *y_i_* ‘resemble’ each other, the stronger X and Y interact. This ‘resemblance’ can be quantified for instance by the cross-correlation. When this is done as a function of frequency, the coherency is determined, which is the most commonly used tool for this purpose. However it has been shown that X and Y can interact, even when *x_i_* and *y_i_* do not ‘resemble’ each other in a simple way. This more general concept, aptly called generalized synchronization, implies that the state of Y is a function of the state of X[22]. Here *x_i_* and *y_i_* do not have to resemble each other, as long as recurrent patterns of *x_i_* coincide (in time) with recurrent patterns of *y_i_*. The synchronization likelihood is a way to quantify this ‘generalized synchronization’. It ranges from a small value close to 0 (P_ref_, no synchronization) to 1 (complete synchronization).

### Data analysis

**Figure 1:**
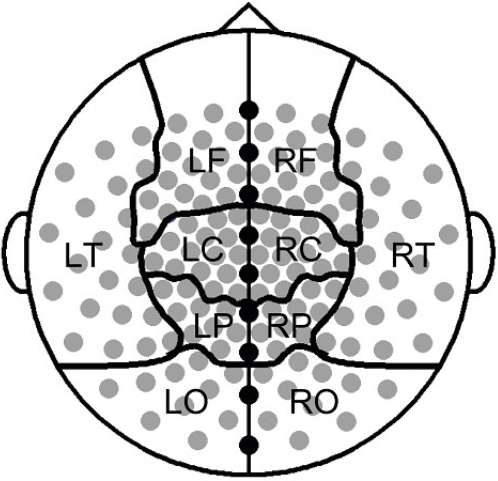
Location of the magnetoencephalographic sensors and their grouping into different sectors.

For the present analysis, 149 of the 151 MEG channels could be used. Signals from two sensors could not be used for technical reasons. MEG recordings of the eyes-closed resting state were converted to ASCII files and down-sampled from 625 to 312.5 Hz. From these ASCII files 4 artifact free epochs of 4096 samples (13.1072 s) per subject were carefully selected by visual analysis (KOD). Computation of synchronization likelihood was done for the following standard frequency bands: delta (0.5–4 Hz), theta (4–8 Hz), low alpha (8–10 Hz), high alpha (10–13 Hz), beta (13–30 Hz) and gamma (30–45 Hz; 55–80 Hz), offline with the DIGEEGXP software developed at the department (CS). Parameter settings for lag *L* and embedding dimension *m* were adjusted per frequency band, as outlined by Montez et al[23]. Following the procedure described by Stam et al[24], *local short distance synchrony* was estimated by averaging the SL values obtained from all pair-wise combinations of channels within each MEG sector (Central, Frontal, Temporal, Parietal and Occipital), in each hemisphere (figure 1). *Interhemispheric synchrony* was estimated by averaging the values of all pair-wise combinations of channels between two MEG sectors (inter-Frontal, inter-Central, inter-Parietal, inter-Occipital and inter-Temporal). Similarly, *intrahemispheric synchrony* was calculated by averaging SL values for all pair-wise combinations of channels between two MEG sectors within each hemisphere: Frontal-Parietal (FP), Frontal-Temporal (FT), Temporo-Occipital (TO) and Parieto-Occipital (PO).

### Statistical analysis

Data are expressed as mean±standard deviation (SD) unless stated otherwise. In the univariate analysis, dichotomous variables were compared using the Fischer's exact test. Since no normal distribution could be assumed for the continuous data, analysis was done by means of the Mann-Whitney *U* test. To reduce the problem of multiple comparisons in the assessment of the SL values, first group differences in total SL averages (taken over all pairs of MEG sensors) per frequency band were tested. Afterwards, only frequency bands with a significant difference in total SL between patient and control group were analyzed in further detail. Spearman's rank correlation coefficient was calculated to study a possible correlation between specific demographic features and synchronization values. All correlations were calculated over the total number of subjects, unless stated otherwise. In all tests, a *p* value <0.05 was accepted as statistically significant. All statistical procedures were carried out by means of the statistical package SPSS 11.0 for Windows (Chicago, Ill, USA).

## Results

### Subject characteristics

Subject characteristics are summarized in table 1. Age and height did not differ between the study groups, whereas bodyweight, BMI and SDS were significantly higher in obese than control subjects. Education level of the obese group consisted of 7 low, 3 middle, and 1 high (according to the Dutch education system). For the control group these numbers were respectively 4, 1 and 3, which were not different (*p* = 0.377, Fischer's exact test).

**Table 1:** Characteristics of the study population.

	obese	control	*P*-value
	n = 11	n = 8	
age, years (±SD)	15.2 (1.5)	16.5 (1.6)	0.125
body height, metres (±SD)	1.66 (0.40)	1.70 (0.83)	0.288
body weight, kilograms (±SD)	107.7 (17.3)	60.4 (6.5)	<0.001
BMI, kg/m2 (±SD)	38.8 (5.7)	21.0 (1.5)	<0.001
SDS BMI (±SD)	3.19 (0.42)	0.14 (0.51)	<0.001

### Synchronization likelihood

#### General outcome

**Figure 2:**
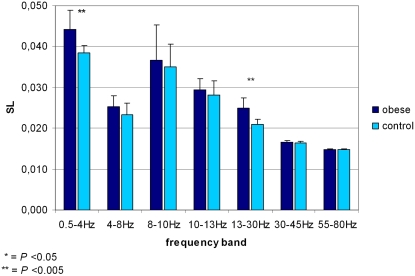
Synchronization values in seven frequency bands in obese and control subjects. Lines on top of the bars represent standard deviations of the means shown. An asterisk indicates a certain significance level in the difference found between the two research groups (Mann-Whitney U).

Total (averaged over all pairs of sensors) synchronization values per frequency band in the obese and control group are illustrated in figure 2. Obese subjects showed consistently higher values for total synchronization than controls. Highly significant differences were found in delta (0.5–4 Hz) and beta (13–30 Hz) band (*P*_delta total_ = 0.001; *P*_beta total_ = 0.002, Mann-Whitney *U* test).

#### Specific frequency bands

**Figure 3:**
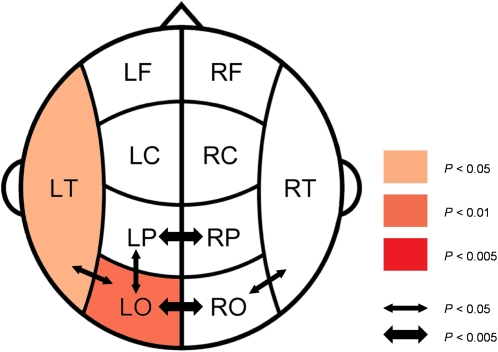
Differences in SL values between obese and control subjects in the delta band. Coloring indicates a significant higher local SL value for the obese compared to the control group; color intensity keeps scale with the significance level of the differences found. Arrows indicate significant higher interhemispheric and long distance SL values for obese compared to control subjects. Here, the thickness of the arrow represents the significance level.

The synchronization differences between obese and control subjects for the delta band are illustrated in more detail in figure 3. The delta band showed increases in local synchrony in left occipital and left temporal areas for the obese as compared to the control group. In interhemispheric and intrahemispheric synchronization, SL values for interoccipital and interparietal, as well as for left parieto-occipital and bilateral temporo-occipital coupling were higher in obese than control subjects.

**Figure 4:**
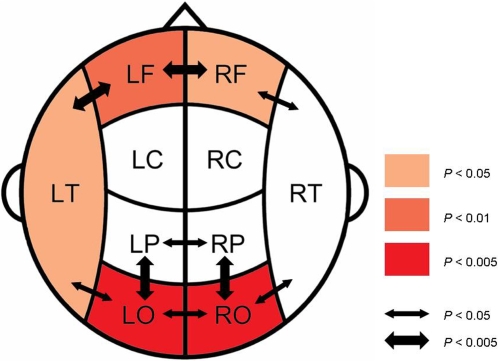
Differences in SL values between obese and control subjects in the beta band. Coloring indicates a significant higher local SL value for the obese compared to the control group; color intensity keeps scale with the significance level of the differences found. Arrows indicate significant higher interhemispheric and long distance SL values for obese compared to control subjects. Here, the thickness of the arrow represents the significance level.

The synchronization differences between obese and control subjects for the beta band are illustrated in figure 4. In the beta band, highly significant differences in SL values between the obese and the control group were located in occipital and frontal regions. Increases in local synchrony for obese as compared to control subjects were present in bilateral occipital and frontal areas and in the left temporal area. In interhemispheric and intrahemispheric synchronization, SL values for interfrontal, interparietal and interoccipital coupling, as well as SL values for bilateral fronto-temporal, parieto-occipital and temporo-occipital coupling were higher in obese than control subjects.

Correlations of total beta and delta SL with age, heart rate and time of data acquisition were explored. No correlation was found between age and SL value (r_delta_ = −0.313, *p* = 0.192; r_beta_ = −0.335, *p* = 0.160), between heart rate and delta SL (r = 0.316, *p* = 0.188) or between acquisition time and beta SL (r = −0.406, *p* = 0.085). A positive correlation was found between heart rate and beta SL (r = 0.709, *p* = 0.001) and negative correlation was found between acquisition time and delta SL (r = −0.467, *p* = 0.044). After adjusting for partial correlation influence of BMI, both correlations turned non-significant (delta SL & acquisition time: r = −0.318, *p* = 0.199; beta SL & heart rate: r = 0.445, *p* = 0.064). In addition, if only data of the obese subjects were taken into account, heart rate did not show a correlation with SL rates (r = 0.569, *p* = 0.067).

## Discussion

The present study is the first to show that functional connectivity in the brain, as assessed by MEG in an eyes-closed resting state, differs between obese and lean adolescents. Several functional imaging studies have been performed both in lean adults and children to investigate the neural response to food stimuli[13], [25], as well as in comparison with obese subjects[26], [27]. Although these studies have contributed to a greater understanding of food-related processes in subcortical structures, they focussed predominantly on evoked alterations in a stimulus-response concept instead of non-task related strength of functional coupling between brain areas.

Altered resting-state functional connectivity is a well known phenomenon in several pathological brain conditions such as Alzheimer's and Parkinson's disease [24], [28]–[30]. In these conditions, possible substrates and pathophysiological explanations for changes in brain functioning have been revealed, but a precise explanation for the specific alterations in functional connectivity still remains unclear. Therefore, in our research concerning obesity we can not fully explain our results.

Electrophysiological alterations in resting-state brain activity have been described extensively in studies performed on substance abuse, such as alcohol and cocaine dependency[31]–[34]. In these pathological conditions, involvement of the brain reward circuitry is a universal feature. The results displayed in these studies suggest that increased electrophysiological activity in the human brain could be correlated to disturbed, and possibly disinhibited motivational cerebral pathway signalling. Since in obesity these pathways appear to be involved as well, our results fit in this concept. It is important to realize that an increase in synchronization does not necessarily imply better functional connectivity though, since optimal brain functioning requires a proper balance between synchronization and desynchronization[35], [36].

Another possible explanation for our results is the recently demonstrated difference in brain composition between obese and lean persons. Assessing gray and white matter structure in obese and non-obese persons using MRI, Haltia et al.[37] discovered white matter expansion in obese compared to control subjects in several brain regions, in particular in the temporal lobes. It has been suggested that this could be a reflection of abnormal lipid metabolism and increased accumulation of fat in central myelin throughout the brain. White matter is involved in the communication between neuronal populations in distinct areas of the brain. The synchronization differences we found between obese and non-obese persons could therefore be the functional output of an altered substrate such as white matter expansion. However, the MEG synchronization showed a rather diffuse pattern of increased functional connectivity extending beyond the areas where increased white matter volume has been observed. Future studies are necessary to explore the potential relationship between increases in resting-state functional connectivity and white matter volume.

Assessing the effect of hyperinsulinemic states on cerebrocortical activity, Tschitter et al [38], [39] were the first to use magnetoencephalography in the study of obesity. In accordance with our findings, they reported involvement of beta band activity. However, in these studies different procedures and analyses were used. For example, beta activity was only directly compared between different conditions within the subject groups. Futhemore, no specific information about the actual spectral properties or precise cortical locations was presented. In the light of these findings, the hyperinsulinemic state in the obese adolescents in the present study, may lead to speculations that insulin resistance, estimated by HOMA, has an effect on functional connectivity of the cerebral cortex.

Of the possible confounding factors that might explain the results, age, pubertal stage and education level, as well as acquisition time did not differ significantly between our two research groups. We also took into account the position of each participants head in the MEG helmet, since a different head position in this helmet might produce systematically different data. During recordings sessions, head coordinates were determined. No significant differences between obese and controls were found. In order to detect a possible different arousal state of all the participants during recording sessions, we measured heart rate. Although obese subjects had a higher heart rate than controls, no correlation with delta band SL values was found. The correlation of heart rate with beta band SL was mainly influenced by body weight itself, and this feature has been reported extensively in the past. Obesity is associated with impaired cardiovascular autonomic function, resulting in higher heart rate values[40], [41]. In our study, it is probably not heart rate itself influencing the oscillatory rhythm, but the large difference in body weight (resulting in higher heart rate) between the two research groups.

Excessive synchronization in delta and beta bands in obesity could be the functional output of disturbed motivational pathways, an altered substrate as white matter expansion or metabolic processes like hyperinsulinemia. In a study where MEG and MRI are used in the same group of subjects, more information could be gathered to explore the association between functional disturbances and structural alterations in the obese brain. In a longitudinal study, the possible effects of weight gain and weight loss on brain structure and brain communication processes could be followed, in order to investigate the (ir)reversibility of synchronization changes.

In summary, we found increased resting-state functional connectivity in the brains of obese adolescents, compared to controls, indicating that functional coupling between brain areas is changed in obesity. Cortical communication processes make an important contribution to cognitive functioning, and our research strengthens the hypothesis that obesity may have an important impact on human brain function.
